# A real-world study to evaluate the safety and efficacy of three injectable neurokinin-1 receptor antagonist formulations for the prevention of chemotherapy-induced nausea and vomiting in cancer patients

**DOI:** 10.1007/s00520-022-07082-7

**Published:** 2022-05-02

**Authors:** George Dranitsaris, Mehdi Moezi, Kate Dobson, Robert Phelan, Sibel Blau

**Affiliations:** 1 Health Economics and Outcomes Research Unit, Quality Care Cancer Alliance, 1624 S. “I” Street, Suite 305, Tacoma, WA 98405 USA; 2 Department of Public Health, Falk College, Syracuse University, Syracuse, NY USA; 3 Cancer Specialists of North Florida, Jacksonville, FL USA; 4 Northwest Medical Specialties, Tacoma, WA USA

**Keywords:** Fosaprepitant, Nausea, Vomiting, Chemotherapy, Infusion reactions

## Abstract

**Background:**

Three different injectable neurokinin-1 (NK-1) receptor antagonist formulations (CINVANTI® [C] vs. intravenous Emend® [E] vs. generic formulations of fosaprepitant [GFF]) were compared with respect to nausea and vomiting control, use of rescue therapy, and the development of infusion reactions over multiple cycles of chemotherapy.

**Methods:**

A retrospective analysis from 17 community oncology practices across the USA was conducted on patients who received moderately or highly emetogenic chemotherapy. The co-primary endpoints were the control of chemotherapy-induced nausea and vomiting (CINV) from days 1 to 5 over all cycles and the frequency of infusion-related reactions. Propensity score weighted multivariable logistic regression analysis was used to compare complete CINV control, the use of rescue therapy, and the risk of infusion reactions between groups.

**Results:**

The study enrolled 294 patients (C = 101, E = 101, GFF = 92) who received 1432 cycles of chemotherapy. Using CINVANTI® as the reference group, comparative effectiveness was suggested in CINV control over all chemotherapy cycles (odds ratio (OR): E vs. C = 1.00 [0.54 to 1.86] and GFF vs. C = 1.12 [0.54 to 2.32]). However, use of rescue therapy was significantly higher in the EMEND® group relative to CINVANTI® (OR = 2.69; 95%CI: 1.06 to 6.84). Infusion reactions were also numerically higher in the EMEND® group, but the difference did not reach statistical significance (OR = 4.35; 95%CI: 0.83 to 22.8).

**Conclusions:**

In this real-world analysis, patients receiving CINVANTI® had a reduced need for CINV rescue therapy and a numerically lower incidence of infusion reactions.

**Supplementary Information:**

The online version contains supplementary material available at 10.1007/s00520-022-07082-7.

## Introduction

Tremendous progress was made towards the control of chemotherapy-induced nausea and vomiting (CINV) following the approval of the 5HT-3 receptor antagonist antiemetics [1, 2]. Patient care was further enhanced with the development of oral aprepitant, the first neurokinin-1 (NK-1) receptor antagonist [3, 4]. The NK-1 receptor antagonists are effective agents for the prevention of CINV when added to standard antiemetic therapy, with multiple supportive studies [5]. Indeed, a recent meta-analysis of randomized trials determined that the addition of NK-1 receptor antagonists to standard therapy improved complete emesis control by approximately 5% in the acute phase (first 24 h) and by 15% in the delayed phase (days 2 to 5) of CINV [6]. Given the supportive data, the guidelines of the American Society of Clinical Oncology consider the NK-1 receptor antagonists to be a standard of care for patients receiving MEC or HEC [7].

Following the initial approval of oral aprepitant in 2003, an intravenous (IV) formulation was developed (EMEND®), which consists of fosaprepitant, a prodrug which is metabolized into aprepitant following IV administration [4, 8]. EMEND® IV 150 mg is administered on day 1 only as an infusion over 20–30 min, approximately 30 min prior to chemotherapy and was demonstrated to be non-inferior to oral aprepitant in patients receiving highly emetogenic chemotherapy [9–11]. In 2019 following the loss of patent protection, several generic formulations of IV EMEND® were also approved in the USA [12, 13].

One of the challenges associated with fosaprepitant is infusion site reactions, with rates ranging from 7% to as high as 67% being reported retrospective studies [14–17]. Characteristics of infusion site reactions from fosaprepitant include pain, erythema, and extravasation [14, 15]. Risk factors for the development of infusion site reactions upon multivariate analysis consist of younger patients, location of IV line (hand vs. forearm), and simultaneous maintenance IV fluid rate < 100 mL/h during the infusion [17].

CINVANTI® is a polysorbate 80-free injectable emulsion of aprepitant that was approved in 2017 by the US FDA for the prevention of acute and delayed CINV following moderately (MEC) or highly emetogenic (HEC) chemotherapy. The approval was based on the results of two randomized, crossover studies in healthy volunteers which demonstrated that CINVANTI® was bioequivalent to IV EMEND® [18]. In addition, it was revealed that subjects who received CINVANTI® had fewer side effects, including infusion site reactions compared to IV EMEND®. One of the potential factors associated with a reduced frequency of infusion site reactions could be the elimination of polysorbate 80 from the CINVANTI® formulation. It has been suggested that polysorbate 80 may contribute to reactions such as pain, erythema, and phlebitis [19, 20].

Given the growing utilization of CINVANTI® since its approval, it would be of interest to compare its efficacy and safety in terms of emesis control and infusion site reactions to IV EMEND® and generic formulations of fosaprepitant in a real-world setting. In this study, cancer patients receiving MEC and HEC within the Quality Cancer Care Alliance Network (QCCA) of practices in the USA were identified and used for a comparative analysis of safety and efficacy between CINVANTI®, IV EMEND®, and generic formulations of fosaprepitant. The primary objectives of the current study were two-fold; to compare nausea and vomiting control between the NK1 alternatives over multiple cycles of MEC and HEC. Secondly, to compare the frequency of infusion reactions between the alternative agents when used during MEC and HEC.

## Methods

### Number of patients

This was a real-world retrospective observational study consisted of 294 adult cancer patients who received MEC or HEC regimens between January 1, 2017 and December 1, 2020. All patients received treatment through the QCCA, a network of 17 community oncology clinics across the USA. A randomized comparative trial between the agents of interest is unlikely to be undertaken. Hence, the only way to evaluate comparative safety and efficacy is through a real-world study. To be entered into the study, patients must have been 18 years of age or older, had received either a MEC or HEC regimen, and had an antiemetic regimen containing a 5HT3 receptor antagonist. In addition, patients must have received CINVANTI®, IV EMEND®, or generic fosaprepitant as part of routine clinical practice and according to institutional administration guidelines, which consisted of a 30-min infusion in normal saline or a 5% dextrose solution. Patients were excluded if they were receiving concurrent radiotherapy, had mechanical risk factors for nausea (i.e., intestinal obstruction), or were receiving chronic treatment with corticosteroids. In addition, patients with chronic nausea and vomiting from other causes such as brain metastases and chronic constipation were also excluded. All 17 community oncology clinics contributed patients into the study, provided the inclusion criteria were met.

### Data collection

Prior to the start of chemotherapy, data collection consisted of patient demographics, disease and treatment characteristics, Eastern Cooperative Oncology Group (ECOG) performance status, and existing comorbidities as assessed by the Charlson comorbidity index [21]. From the first cycle until the completion of chemotherapy, data was collected on the type of chemotherapy, type and dose of antiemetics used, the development of infusion site reactions, the type of reaction (e.g., hives, swelling), grade, location, the dosage and dilution of the NK1-antagonist received, the location of the IV line (forearm vs. hand vs. antecubital fossa), and the simultaneous maintenance IV fluid rate (< 100 vs. ≥ 100 mL/h) during the NK1-antagonist infusion. Data abstraction also includes the number of vomiting episodes, the occurrence, intensity, and duration of nausea in the first 24 h and up to day 5 following chemotherapy and the use of rescue medication for breakthrough emesis. In addition, the total number of cycles delivered, as well as the number of hospitalizations, visits to an emergency department, unscheduled clinic visits and resource utilization for patient supportive care (e.g., hydration) secondary to poorly controlled CINV were also collected. The study was not initiated until the protocol was reviewed and approved by the QCCA Ethics Review Committee. Given the retrospective nature of the data collection, patient informed consent was not required.

### Sample size and statistical considerations

The co-primary endpoints in this comparative effectiveness analysis were the frequency of infusion site reactions and complete CINV response over all cycles of chemotherapy. CINV complete response was defined as no vomiting, no retching, no clinically documented moderate or severe nausea, and no need for rescue therapy. Secondary endpoints consisted of the use of rescue therapy and health care resource use to manage poorly controlled CINV. The target sample size for this study was 100 patients per group, for an overall sample size of 300 patients receiving either MEC or HEC chemotherapy. The incidence of infusion site reactions among all peripherally administered doses of IV EMEND® was reported from one single-institution study in the USA to be 15% [17]. With 100 patients in each group, the study had a 77% power to identify an 85% relative risk reduction (i.e., an odds ratio = 0.15) in the incidence of infusion site reactions in patients who received CINVANTI® over multiple cycles of chemotherapy. Given that two comparisons were undertaken, the type I error probability was set to 0.025 using Fisher’s exact test to evaluate the null hypothesis of no difference in the rate of infusion site reactions between CINVANTI® and the two alternatives.

Demographic data, anticancer therapy, antiemetic prophylaxis, and all clinical outcomes’ data were presented as descriptive statistics as means, medians, or proportions. One of the challenges associated with observation studies (unlike randomized trials) is patient selection bias. Systematic factors such as patient self-selection, clinician selection, or geographic selection can lead to patients receiving one therapy preferentially over another. Such bias can affect the results of any comparative analysis in an observational study. To address this issue, patient groups need to be adjusted for the possible impact of this selection bias.

Propensity score analysis is two-phase technique used to estimate a treatment effect in comparative groups selected by non-random means [22, 23]. In the first phase of propensity score analysis, variables that influence group selection are used to model the probability of receiving treatment (or of being in the reference group in this case, the CINVANTI® group). The final model is then used to generate a probability, which is called the propensity score. In the second phase, the propensity score is used to adjust for pre-existing group differences in the evaluation of outcomes under investigation [22–24]. There are several ways to use of propensity scores, such as matching patients based on their propensity score or their use as a weighting variable during a multivariate analysis. Assuming that all relevant covariates are included in the propensity score model, the group effect size observed in a propensity score analysis represents an unbiased estimate of the true treatment effect under randomized trial conditions [22, 23].

In the current study, any covariate with a marginal association with membership in the CIVANTI group (*p* < 0.10) (vs. the two other groups) was considered for inclusion in the propensity score model. A main-effects multivariate nominal logistic regression model was developed using variables identified in the previous step and retained following a backwards elimination process with *p* < 0.10. Nominal logistic regression is the recommended approach for estimating propensity scores because the current study is composed of three comparative groups. The final propensity scores were then used in the subsequent analyses.

To evaluate the primary and secondary endpoints, propensity score weighted multivariable logistic regression analysis for repeated measures, with an adjustment for clustering on the patient, was used to compare IV EMEND® and generic fosaprepitant to CINVANTI® (reference group) over repeated cycles of chemotherapy. The models also contained patient demographics or clinical characteristics at baseline that were associated with infusion site reactions and complete CINV control. Independent variables with a *p* < 0.05 will be retained in the final model via a backwards elimination process. All of the statistical analyses were performed using Stata, release 16.0 (Stata Corp., College Station, TX, USA).

## Results

Over the evaluation period, a total of 294 patients received one of the three NK1-inhibitor antagonist formulations. Patient groups were reasonably well balanced for an observational study in terms of age, BMI, disease stage, and Charlson comorbidity score (Table 1). However, there was group imbalance with respect to female sex, race, cancer diagnosis, disease stage, performance status, and prevalence of active diabetes (Table 1). A comparison of treatment characteristics also suggested imbalance in the administration of MEC/HEC qualifying agents. Indeed, 40.6% (41 of 101) of CINVANTI® received carboplatin compared to 34.6% and 20.6% in the IV EMEND® and generic fosaprepitant groups, respectively (Table 2). Overall, patients in the CINVANTI® received a total of 495 cycles of chemotherapy, compared to 458 and 479 cycles in patients who were treated with IV EMEND® and generic fosaprepitant. This translated to a median of 3 cycles of MEC/HEC regimens delivered among the three groups (Table 2).

**Table 1:** Characteristics of patients between groups

Characteristic (median, range)	Cinvanti (*n* = 101)	Emend IV (*n* = 101)	Generic alternatives (*n* = 92)
Patient age	60 (21 to 82)	59 (22 to 82)	62 (25 to 80)
Female sex	77.2%	81.2%	62.0%
BMI	28.5 (16.2 to 49.6)	29.7 (16.2 to 52)	29.0 (16.6 to 48)
Race			
White	69.3%	80.2%	69.6%
Black	10.9%	4.0%	4.4%
Other	5.0%	3.0%	1.1%
Not documented	14.8%	12.9%	25.0%
Cancer diagnosis			
Breast	43.6%	49.5%	31.5%
Lung	12.9%	8.9%	13.0%
Gynecological	14.8%	14.8%	2.3%
Colorectal	10.9%	5.0%	16.3%
Other	17.8%	21.8%	36.9%
Disease stage			
I–III	67.3%	72.3%	64.1%
IV	21.8%	20.8%	26.1%
Not documented	10.9%	6.9%	9.8%
ECOG			
0	47.5%	55.4%	63.0%
1	32.7%	31.7%	27.2%
≥ 2	5.9%	7.9%	3.3%
Not documented	13.9%	5.0%	6.5%
Charlson comorbidity score	3 (2 to 10)	3 (2 to 13)	4 (2 to 16)
Active diabetes^1^	11.9%	10.9%	26.1%
Prior fosaprepitant	4.0%	2.0%	2.2%

Abbreviations: *IV* intravenous, *BMI* body mass index, *ECOG* Easter Cooperative Oncology Group performance status

^1^Define as patients who were receiving anti-diabetic medication

**Table 2:** Treatment characteristics prior to the start of chemotherapy

Characteristic (median, range)	Cinvanti (*n* = 101)	Emend IV (*n* = 101)	Generic alternatives (*n* = 92)
MEC/HEC agent			
Carboplatin	40.6%	34.6%	20.6%
Cisplatin	4.0%	6.9%	10.9%
Oxaliplatin	12.9%	7.9%	19.6%
Doxorubicin	36.6%	43.6%	41.3%
Number of cycles (median)	3 (1 to ≥ 8)	3 (1 to ≥ 8)	3 (1 to ≥ 8)
Total number of cycles given	495	458	479
Vesicant part of treatment	55.4%	57.4%	47.8%
Number of vesicants (median)	1 (0 to 2)	1 (0 to 2)	0 (0 to 2)
NK1 dose	130 mg	150 mg	150 mg
CVA for NK1 infusion	97.0%	86.1%	94.6%
NK1 infusion duration			
20 min	5.9%	11.9%	40.2%
30 min	42.6%	56.4%	14.1%
40 min	3.0%	4.0%	1.1%
Other	48.5%	27.7%	44.6%
IV fluid rate			
< 100 mL/h	33.7%	14.8%	6.5%
≥ 100 mL/h	40.6%	65.4%	58.7%
Not documented	25.7%	19.8%	34.8%
Dexamethasone given	97.0%	100%	100%
Dexamethasone given IV	97.0%	99.0%	99.0%
Dexamethasone dose (median)	10 mg (1 to 20)	12 (4 to 40)	12 (10 to 12)
Dexamethasone added to IV bag	16.8%	11.9%	52.2%
Palonosetron used	90.1%	91.0%	95.6%

Abbreviations: *5HT3* serotonin 5-HT_3_ receptor antagonist, *NK1* neurokinin 1 receptor antagonist, *CVA* central venous access, *IV* intravenous, *MEC* moderately emetogenic chemotherapy, *HEC* highly emetogenic chemotherapy

The use of vesicants as part of the regimen was then examined. Approximately 55.4% of the 495 CINVANTI® supported cycles consisted of a vesicant compared to 57.4% and 47.8% in the IV EMEND® and generic fosaprepitant groups. The dose of the respective NK1 receptor antagonist formulations was consistent with the respective product monographs, namely, 130 mg for CINVANTI® and 150 mg for the two alternatives (Table 2). However, an unexpected finding was that the NK1 infusion was delivered via a central line in over 85% of all chemotherapy cycles. There was also variability in the duration of the NK1 infusion and the associated IV fluid rate (Table 2). In terms of ancillary antiemetic prophylaxis, at least 97% of all cycles were supported with intravenous dexamethasone, with median doses ranging from 10 to 12 mg. However, it was noted that in the generic fosaprepitant group, the dexamethasone was added to the infusion bag in 52.2% of cycles compared to only 16.8% and 11.9% for the CINVANTI® and EMEND® supported cycles, respectively. Lastly, palonosetron was the 5HT-3 receptor antagonist of choice in over 90% of cycles across the three groups (Table 2).

The assessment of efficacy began with an unadjusted comparison of complete CINV response in the first 24 h following chemotherapy, days 2 to 5 and days 1 to 5. Overall, there was excellent day 1 CINV control in each of the three groups, ranging from 91% with IV EMEND® to 98.1% with generic formulations of fosaprepitant. Control of delayed CINV from days 2 to 5 was also excellent, with CINVANTI® and generic fosaprepitant formulations having a numerical advantage over IV EMEND® (Table 3). Indeed, when the results were assessed from days 1 to 5, IV EMEND® appeared to have the lowest CINV response rate compared to the other two groups. This was also suggested by an evaluation of CINV response over the first six cycles of MEC/HEC (Supplemental Fig. 1). The modestly reduced efficacy with IV EMEND® was also consistent with the use of rescue therapy across the three groups. Approximately 11.8% of patient cycles in the IV EMEND® required rescue therapy compared to only 4.2% and 3.1% of cycles that were supported by CINVANTI® and generic fosaprepitant formulations (Table 3). Over the first six cycles of chemotherapy, between 10 and 15% of IV EMEND® patients received rescue therapy compared to less than 10% in the other two groups (Supplemental Fig. 2).

**Table 3:** Unadjusted efficacy and safety outcomes following all cycles of chemotherapy

Outcomes over all cycles (median, range)	Cinvanti (*n* = 495)	Emend IV (*n* = 458)	Generic alternatives (*n* = 479)
Complete CINV response^1^: day 1	92.1%	91.0%	98.1%
Complete CINV response^1^: days 2 to 5	90.1%	84.1%	93.7%
Complete CINV response^1^: days 1 to 5	87.9%	82.3%	92.5%
Documented use of rescue therapy	4.2%	11.8%	3.1%
Infusion site reactions	0.4% (2)	1.5% (7)	1.2% (6)
Reaction grade	*N* = 2	*N* = 7	*N* = 6
Grade 1	1	4	3
Grade 2	1	0	2
Unable to grade	0	3	1
Characteristics of reaction			
Redness/itching	1	3	5
Rash	1	0	1
Edema	1	2	1
Hives	0	1	0
Infusion site pain	1	3	2
Swelling	1	3	1
Extravasation	0	1	1

Abbreviations: *5HT3* serotonin 5-HT_3_ receptor antagonist, *NK1* neurokinin 1 receptor antagonist, *IV* intravenous, *CINV* chemotherapy-induced nausea and vomiting

^1^Complete CINV response defined as no significant nausea, no vomiting, and no documented use of rescue therapy

The analysis was continued with an assessment of infusion site reactions. Overall, there were few documented events, with only 2 reactions (0.4%) identified in the CINVANTI® group compared to 7 (1.5%) and 6 (1.2%) in the IV EMEND® and generic fosaprepitant group, respectively (Table 3). The reactions were either grade 1 or 2 and characterized by redness, itching, rash, edema, swelling, and infusion site pain. The reactions were managed with supportive interventions and in no case was the reaction severe enough to require a permanent discontinuation of the NK-1 receptor antagonist (Table 3).

The propensity scores derived for each patient were then used to perform a weighted multivariable logistic regression analysis on the primary and secondary study endpoints. Using complete CINV response from day 1 to day 5 as the dependent variable and designating CINVANTI® as the reference group, the findings suggested comparable efficacy between the three groups, with the 95%CI of the odds ratios (OR) encompassing 1.0 (Table 4). The findings also identified independent risk factors for reduced CINV response. Control of CINV was significantly reduced in cycle 2 and beyond (i.e., there was better control in cycle 1), in breast cancer patients, females and when the IV fluid rate was set to ≥ 100 mL/h. In contrast, there was a dramatic and statistically significant improvement in complete CINV response when dexamethasone was added to the IV bag (OR = 6.42: 95%CI: 3.1 to 13.5).

**Table 4:** Propensity score weighted analysis on complete CINV response from days 1 to 5

Variables^1^	Odds ratio^2^	95%CI	Impact on CINV complete response
NK1 group (vs. CINVANTI®)			
IV EMEND®	1.00	(0.54 to 1.86)	NS
Generic alternatives	1.12	(0.54 to 2.32)	NS
Cycle 1 vs. ≥ cycle 2	0.44	(0.29 to 0.55)	↓ likelihood by 56%
Breast vs. other cancers	0.27	(0.16 to 0.48)	↓ likelihood by 73%
Female sex	0.37	(0.14 to 0.95)	↓ likelihood by 63%
IV fluid rate ≥ 100 mL/h	0.20	(0.10 to 0.37)	↓ likelihood by 80%
Dexamethasone added to IV bag	6.42	(3.1 to 13.5)	↑ likelihood by 6.42 times
Adjusted *R*^2 statistic^3^	23.6%		

Dependent variable: complete control of CINV from days 1 to 5 post chemotherapy

Abbreviations: *NK1* neurokinin 1 receptor antagonist, *CINV* chemotherapy-induced nausea and vomiting, *NS* not significant

^1^These are the final variables that were retained following the application of the likelihood ratio test (*p* < 0.05 to retain) in a backwards elimination process

^2^An odds ratio of less than one indicates lower likelihood and greater than one an increased likelihood

^3^This is the proportion of variability in the dependent variables than is accounted for by the independent variables

The propensity score weighted analysis was continued with an evaluation of the dependent variable, need for rescue therapy. Using the CINVANTI® group as the reference, there was no statistically significant difference in the need for rescue therapy when compared to patients who received generic fosaprepitant (Table 5). In contrast, patients who received IV EMEND® were approximately 2.69 times more likely over all cycles of chemotherapy to require rescue therapy for breakthrough nausea and vomiting (OR = 2.69; 95%CI: 1.06 to 6.84). When risk factors were evaluated in the logistic regression model, cycle 2 and beyond and the use of carboplatin were associated with a significantly higher need for rescue therapy (Table 5).

**Table 5:** Propensity score weighted analysis on the use of rescue therapy from days 1 to 5

Variables^1^	Odds ratio^2^	95%CI	Impact on need for rescue therapy
NK1 group (vs. CINVANTI®)			
IV EMEND®	2.69	(1.06 to 6.84)	↑ likelihood by 2.7 times
Generic alternatives	0.68	(0.22 to 2.07)	NS
Cycle 1 vs. ≥ cycle 2	2.04	(1.11 to 3.73)	↑ likelihood by 2.0 times
Carboplatin given	2.68	(1.07 to 6.70)	↑ likelihood by 2.7 times
Patient age	0.96	(0.93 to 0.98)	↓ likelihood in older patients
Adjusted *R*^2 statistic^3^	10.8%		

Dependent variable: documented use of rescue therapy

Abbreviations: *NK1* neurokinin 1 receptor antagonist, *CVA* central venous access, *CINV* chemotherapy-induced nausea and vomiting

^1^These are the final variables that were retained following the application of the likelihood ratio test (*p* < 0.05 to retain) in a backwards elimination process

^2^An odds ratio of less than one indicates lower likelihood and greater than one an increased likelihood

^3^This is the proportion of variability in the dependent variables than is accounted for by the independent variables

The second dependent variable evaluated under a propensity weighted multivariable logistic regression model was infusion site reactions. Over all cycles of chemotherapy, patients who received IV EMEND® had an increased risk of infusion site reactions compared to patients in the CINVANTI® group (OR = 4.35; 95%CI: 0.83 to 6.84), but the incremental risk failed to reach statistical significance, likely due to the small number of events (Table 6). Statistically significant risk factors for infusion site reactions identified in the analysis consisted of active diabetes, the use of oxaliplatin, and the number of vesicants that were part of the anticancer protocol. Indeed, increasing the number of vesicants from one to two had a substantial impact on the risk of reactions, with the OR (vs. no vesicants) increasing from 5.82 to 8.70 (Table 6). In contrast, delivering the NK1 via a central line was protective, reducing the risk of infusion site reactions by approximately 75% (*p* < 0.05).

**Table 6:** Propensity score weighted analysis on the frequency of infusion reactions

Variables^1^	Odds ratio^2^	95%CI	Impact on reaction risk
NK1 group (vs. CINVANTI®)			
IV EMEND®	4.35^4^	(0.83 to 22.8)	↑ 4.4 times
Generic alternatives	1.15^5^	(0.21 to 6.38)	NS
Active diabetes	8.7	(2.68 to 28.3)	↑ 8.7 times
Number of vesicants (vs. none)			
One	5.82	(2.02 to 16.8)	↑ 5.8 times
Two	8.70	(2.23 to 33.9)	↑ 8.7 times
Oxaliplatin part of treatment	9.72	(3.74 to 25.3)	↑ 9.7 times
NK1 given via CVA	0.25	(0.65 to 0.98)	↓ by 75%
Adjusted *R*^2 statistic^3^	16.6%		

Dependent variable: infusion reactions of any grade

Abbreviations: *NK1* neurokinin 1 receptor antagonist, *CVA* central venous access

^1^These are the final variables that were retained following the application of the likelihood ratio test (*p* < 0.05 to retain) in a backwards elimination process

^2^An odds ratio of less than one indicates lower risk and greater than one increased risk

^3^This is the proportion of variability in the dependent variables than is accounted for by the independent variables

^4^The *p* value for the OR between Emend and Cinvanti was *p* = 0.082

^5^The *p* value for the OR between generic fosaprepitant and Cinvanti was *p* = 0.87

The final parameter evaluated was health care resource use for poorly controlled CINV over all cycles of chemotherapy. Over 495 cycles of chemotherapy supported by CINVANTI®, there were 22 instances where patients required hydration with IV fluids. In the IV EMEND® and generic fosaprepitant groups where 458 and 479 chemotherapy cycles were delivered, hydration was required in 18 and 16 occasions, respectively. In addition, there were 7 visits to emergency rooms for poorly controlled emesis in the CINVANTI® and 7 visits in the IV EMEND® group, compared to 5 visits in the generic control group. None of the differences in health care resource use for poorly controlled CINV reached statistical significance.

## Discussion

Observational data from real-world experiences can be an effective source for evidence generation and may provide important clinical insights that may not have been detected in randomized controlled trials [25]. A comparative analysis of safety and efficacy using real-world data was undertaken to evaluate CINVANTI® as an alternative to IV EMEND® and generic formulations of fosaprepitant, which are now widely available in many countries worldwide [12, 13]. The findings indicated comparable efficacy in terms of complete CINV response throughout all cycles of chemotherapy. However, breakthrough CINV occurred more frequently with IV EMEND®, which was characterized by significantly higher use of rescue therapy. Indeed, the need for rescue therapy was two to three times higher in patients who received IV EMEND® compared to the CINVANTI® group.

One of the challenges with using observational data for comparative effectiveness research is the results may not be externally valid when applied to other practice settings. The findings of the multivariate analysis on complete CINV response and the need for rescue therapy supported the external validity of the findings. Risk factors retained in the regression models consisted of cycle number, female sex, and patient age. Poor CINV control and the need for rescue therapy were highest in the first cycle of chemotherapy. Younger patients also had an increased need for rescue therapy, while female patients had a reduced likelihood of achieving a complete CINV response. The first cycle of chemotherapy, younger age, and female gender have been reported as risk factors for CINV in multiple studies [26, 27]. Two other significant factors identified in the multivariate analysis were the impact of the IV fluid rate and adding dexamethasone to the infusion bag. Patients whose IV fluid rate was ≥ 100 mL/h were 80% less likely to achieve complete CINV control. Therefore, reducing the IV fluid rate to less than 100 mL where clinically possible could have a profound effect of CINV control. It was also discovered that adding dexamethasone to the IV bag can have a large impact on attaining CINV control, with an odds ratio of 6.42. This was an unexpected finding and deserves some speculative commentary. It is possible that in a busy infusion clinic, where the dexamethasone has not been added to the IV bag, health care providers may at times forget to administer the drug. This is one possible explanation, as such a large effect size by the simple addition dexamethasone to the IV bag would not be expected. However this is only speculation and further inquiry into antiemetic administration policies within the participating practices is warranted. Nevertheless, cancer centers should consider a policy where dexamethasone be routinely added to the antiemetic IV infusion bag because it would eliminate the possibility of the drug being omitted.

The second co-primary endpoint evaluated was infusion site reactions. Contrary to what has been reported in the literature [14, 15, 17], such events were rare, occurring in less than 2% of cycles across all three drug groups. This finding may be related to the observation that the NK1 inhibitors were given via central line in over 85% of cycles in our sample. In contrast, the high reaction rates to IV EMEND® reported in the literature were primarily in patients who received the drug via peripheral venous administration [15–17]. The protective effects of central line administration were suggested by the findings of the multivariate analysis. The administration of the NK1 agent via a central line reduced the risk of infusion reactions by approximately 75% (OR = 0.25). This effect may be due to the large fluid volume in a central line compared to a peripheral line or the size of the venous system being accessed. Therefore, another important policy recommendation derived from the current study is that all NK1 agents be administered centrally, whenever possible. It was also interesting to note that patients with active diabetes, or those who received one or more vesicants were at a considerably higher risk of infusion site reactions. Given these findings, another policy recommendation would be to consider CINVANTI® in such high-risk subgroups.

There are several important limitations in this comparative effectiveness study. This was a retrospective observational study using real-world data and not a randomized trial. Consequently, there were imbalances in some important prognostic factors. Complete CINV control has been reported to be between 60 and 70% in prospective observational studies and randomized trials that evaluated CINV control over multiple cycle of chemotherapy [26, 28]. In this study, complete CINV control was measured from electronic medical records and determined to be between 80 and 100%. It is important to acknowledge that measuring CINV control, particularly nausea from electronic medical records can be challenging. Therefore, it is likely that the measurement of complete CINV control was overestimated because there may have been instances where patient reported nausea and vomiting was not documented in the medical record. Lastly, there are several generic fosaprepitant formulations in use across the QCCA network. Hence, the generic fosaprepitant group was composed of several products, as opposed to a single generic formulation. The source of rescue medication taken by patients was the physician’s notes and the pharmacy prescription records. However, it must be acknowledged that the physician’s notes may not been complete and could underestimate the use of rescue medication. Despite these limitations, the findings of this study appear to be credible and do contribute to the supportive care literature.

In conclusion, the findings from this real-world analysis of community oncology practices in the USA have important clinical implications. The use of CINVANTI® as an alternative to IV EMEND® can reduce the need for rescue therapy and may lower the incidence of infusion site reactions. In addition, it was discovered that patients with active diabetes and those receiving one or more vesicants are at a substantially higher risk of infusion site reactions. Strategies to reduce this risk in such patients may include delivering the NK1 antagonist via a central line or substituting CINVANTI®.

## Supplementary Information

Below is the link to the electronic supplementary material.

Supplementary file1 (DOCX 26 KB)

## Data Availability

The data are available from the primary author for independent replication.

## Code availability

The data are available in Stata V16.0.
