# Supplementary material for: A real-world study to evaluate the safety and efficacy of three injectable neurokinin-1 receptor antagonist formulations for the prevention of chemotherapy-induced nausea and vomiting in cancer patients
Source: Support Care Cancer. 2022 May 2;30(8):6649–58. doi: 10.1007/s00520-022-07082-7 (PMC9213362; doi:10.1007/s00520-022-07082-7)
Supplement: Supplementary file 1 — Supplementary file1 (DOCX 26 KB) [file 520_2022_7082_MOESM1_ESM.docx]

**LIST OF SUPPLEMENTAL FIGURES**

1. CINV complete response over the first six cycles of chemotherapy.
2. Reported need for rescue therapy over the first six cycles of chemotherapy.

###

**Figure 1**. CINV complete response over the first six cycles of chemotherapy. Odds ratio for Emend IV (E) and generic fosaprepitant (GF) vs. Cinvanti (C) = 1.00 (p = 0.98) and 1.12 (p=0.75) respectively.

**Figure 2**. Reported need for rescue therapy over the first six cycles of chemotherapy. Odds ratio for Emend IV (E) and generic fosaprepitant (GF) vs. Cinvanti (C) = 2.69 (p = 0.037) and 0.68 (p=0.50) respectively.
